# Rapamycin Prevents Sulfate-Reducing Bacteria-Induced Effects on Snail and GSK-3 and Impaired Intestinal Barrier

**DOI:** 10.3390/microorganisms14040781

**Published:** 2026-03-30

**Authors:** Sudha B. Singh, Cody A. Braun, Amanda Carroll-Portillo, Henry C. Lin

**Affiliations:** 1 Biomedical Research Institute of New Mexico, New Mexico VA Health Care System, Albuquerque, NM 87108, USA; sbsingh14@salud.unm.edu (S.B.S.);; 2 Division of Gastroenterology and Hepatology, Department of Internal Medicine, University of New Mexico, Albuquerque, NM 87108, USA; 3 Medicine Service, New Mexico VA Health Care System, Albuquerque, NM 87108, USA

**Keywords:** *Desulfovibrio*, Sulfate Reducing Bacteria (SRB), Snail transcription factor, Glycogen Synthase Kinase (GSK-3), intestinal epithelial permeability, rapamycin

## Abstract

*Desulfovibrio* spp. are sulfate-reducing bacteria (SRB) associated with conditions such as inflammatory bowel disease (IBD) that are linked to intestinal barrier dysfunction (leaky gut). Previously, we reported that *Desulfovibrio vulgaris* (DSV) caused increased intestinal permeability by upregulating nuclear transcription factor Snail. However, the signaling mechanisms underlying this effect remain unclear. Glycogen synthase kinase-3 (GSK-3) is a serine/threonine kinase that maintains intestinal barrier integrity and negatively regulates Snail and promotes its degradation by proteasomes. Rapamycin has been shown to protect the intestinal barrier and is also known to activate GSK-3. In this study, we investigated whether DSV disrupts intestinal barrier function through modulation of GSK-3 signaling and whether rapamycin could counteract these effects. Using a previously established DSV-induced paracellular permeability model using polarized Caco-2 monolayers, here, we showed that DSV induced inhibitory phosphorylation of GSK-3. Pretreatment of cells with rapamycin prevented DSV- induced phospho- inactivation of GSK-3, suppressed Snail expression and nuclear localization, and significantly reduced DSV-induced barrier permeability. Inhibition of proteasomal degradation with MG132 abolished the protective effects of rapamycin on barrier permeability, supporting a role for GSK-3–mediated proteasomal regulation of Snail. Together, these findings identify GSK-3 signaling as a novel mechanism underlying DSV-induced intestinal barrier dysfunction and highlight rapamycin as a potential therapeutic approach strategy to protect intestinal barrier integrity in response to DSV. Specifically, targeting the GSK-3/Snail pathway may represent a promising strategy to mitigate SRB-associated intestinal barrier disruption.

## 1. Introduction

Sulfate-reducing bacteria (SRB) are minor members of the resident gut bacterial community that are mainly recognized for their production of hydrogen sulfide (H_2_S), an important gasotransmitter. Owing to numerous reports of their negative effects on host physiology, SRB are often classified as opportunistic pathobionts. Overgrowth of *Desulfovibrio*, the most abundant genus among SRB, is observed in diseases such as inflammatory bowel diseases, metabolic syndrome, and neurodegenerative diseases that are also associated with leaky gut [1]. For many of these diseases, *Desulfovibrio* has been suggested to be the driving factor that contributes to their pathogenesis. We have previously demonstrated that *Desulfovibrio vulgaris* (DSV) induced an increase in tight junction permeability in intestinal epithelial cells and disrupted the localization of tight junction protein (TJP) Occludin [2]. These effects of DSV were dependent on Snail, a nuclear zinc finger transcription factor, as silencing of Snail inhibited DSV-induced increase in barrier permeability. DSV caused an increase in Snail expression and its nuclear translocation. As leaky gut is a feature of many diseases that are associated with SRB overgrowth, our previous study suggested that DSV may play a causal role in pathophysiology of these diseases by inducing leaky gut via Snail.

Glycogen synthase kinase-3 (GSK-3) is a ubiquitous serine/threonine kinase with two isoforms, α and β, that are highly homologous to each other and are encoded by different genes. GSK-3 controls cellular functions such as glycogen metabolism, insulin signaling, cell proliferation, apoptosis, and neuronal function [3]. GSK-3 is involved in various diseases such as cancer, kidney disease, and myocardial disorders [4,5,6]. In the context of barrier function, GSK-3 has been shown to maintain epithelial barrier function by regulating apical tight junction proteins such as Occludin, Claudin-1, and E-cadherin [7]. GSK-3 regulates over 100 targets including the transcription factor Snail [8]. GSK-3β inhibits Snail by causing its phosphorylation and its degradation mainly by the proteasomal pathway [9]. Inhibition of GSK-3 activity is mediated by its phosphorylation at serine position Ser21 in GSK-3α and at Ser-9 in GSK-3β [10,11]. Inhibiting GSK-3 activity by its phosphorylation stabilizes Snail and causes an increase in the nuclear translocation and activity of Snail [12,13].

Rapamycin is a mammalian target of rapamycin (mTOR) inhibitor that has been reported to protect the tight junction barrier in intestinal epithelial cells in various experimental settings [14,15,16]. In these studies, the protective effects of rapamycin are mainly attributed to its induction of autophagy, a conserved eukaryotic degradative machinery known to play a crucial role in health and diseases. Rapamycin and other mTOR inhibitors have also been reported to mediate their protective effects, such as suppressing oncogenic proteins and cancer growth, via activation of GSK-3 kinase [17,18,19]. The objective of this study was to determine whether rapamycin protected against DSV-induced increased intestinal barrier permeability and to examine whether this protection involved modulation of GSK-3 signaling and downstream regulation of the Snail transcription factor.

## 2. Results

### 2.1. Rapamycin Prevents DSV-Induced Increased Intestinal Permeability In Vitro

**Figure 1:**
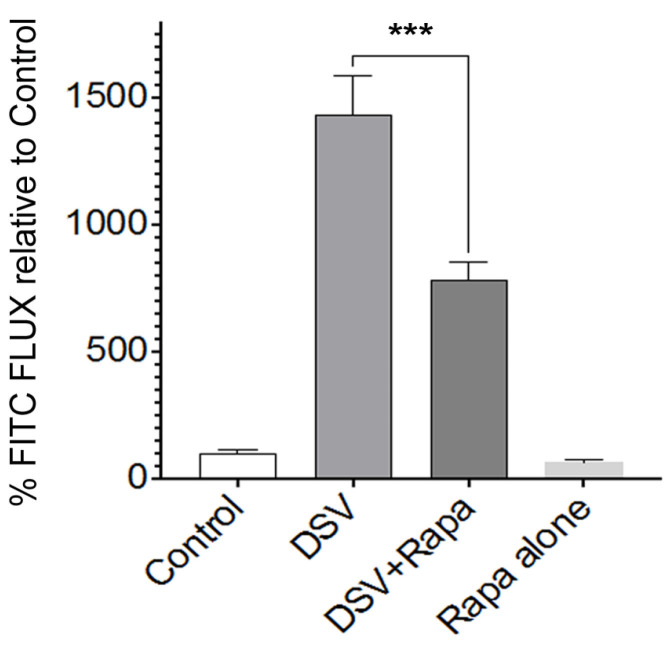
Rapamycin prevents DSV-induced increased intestinal barrier permeability. Polarized Caco-2 monolayers were treated with rapamycin or vehicle prior to DSV infection. Barrier permeability was assessed by measuring apical-to-basolateral paracellular flux of 4 kDa FITC-dextran. Data represent mean ± SEM from at least three independent experiments. *** *p* < 0.001.

First, we tested whether rapamycin inhibited DSV-induced increase in barrier permeability in polarized and differentiated intestinal Caco-2 cells. Cells were treated with rapamycin (50 nm) 2 h before and throughout the infection with DSV for 24 h. Paracellular permeability was assessed by measuring the flux of 4 kDa FITC-dextran through a trans-well chamber as a percentage relative to a control that was set to a value of 100 (Figure 1). Infection with DSV MOI 20 induced a significant increase in FITC flux when compared to the uninfected control (DSV: 1433 ± 50.8 vs. control: 100.0 ± 10.13, *p* < 0.001), which is in keeping with our previous study [2]. However, in cells that were treated with DSV and rapamycin, a significant reduction in FITC flux was observed when compared to DSV-treated cells (DSV + Rapa: 779.3 ± 70.76 vs. DSV: 1433 ± 50.8, *p* < 0.001 compared to DSV). Rapamycin alone did not affect FITC flux when compared to the control (Rapa: 61.43 ± 13.89 vs. control 100.0 ± 10.13, *p* > 0.05). These results suggest that rapamycin significantly inhibited DSV-induced increase in barrier permeability.

### 2.2. Rapamycin Prevents DSV-Induced Snail Protein Expression and Its Nuclear Translocation

**Figure 2:**
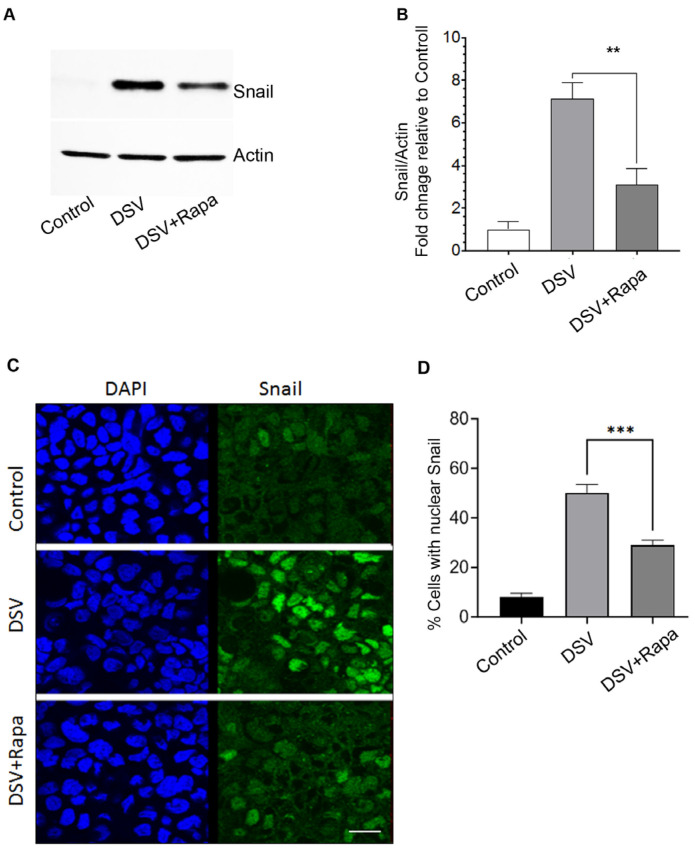
Rapamycin prevents DSV-induced Snail protein expression and its nuclear translocation. (**A**) Representative Western blot showing Snail expression in polarized cells infected with DSV with or without rapamycin pretreatment. Actin was used as a loading control. (**B**) Quantification of Snail protein levels normalized to actin and expressed relative to control using ImageJ/Fiji (2.1.0/1.53c). (**C**) Representative immunofluorescence images showing Snail localization in cells treated with DSV with or without rapamycin pretreatment. Nuclei are stained with DAPI, and Snail is shown in green. (**D**) Quantification of the percentage of cells displaying nuclear Snail staining. Data represent mean ± SEM from at least three independent experiments. ** *p* < 0.01; *** *p* < 0.001. Scale bar = 20 μm.

DSV induces Snail expression and promotes its nuclear translocation in intestinal epithelial cells [2]. As Snail is responsible for disrupting barrier permeability in response to DSV, we asked whether rapamycin inhibited stimulatory effects of DSV on Snail. Cells were pretreated with rapamycin followed by DSV challenge and processed for Western blotting to assess Snail protein expression (Figure 2A). As expected, DSV induced Snail protein expression in Caco-2 cells when compared to control cells (DSV: 7.14 ± 0.73 vs. control: 1.00 ± 0.37). However, the presence of rapamycin inhibited DSV-induced upregulation of Snail (DSV + Rapa: 3.10 ± 0.74 vs. DSV: 7.14 ± 0.73, *p* < 0.01 compared to DSV, Figure 2A,B). Next, we analyzed the effects of rapamycin on DSV-induced nuclear translocation of Snail by comparing the percentage of cells positive for nuclear Snail between treatment groups (Figure 2C,D). In keeping with our previous report, DSV induced nuclear localization of Snail when compared to uninfected control cells (DSV: 69.07% ± 0.16 vs. control: 4.70% ± 2.75, *p* < 0.001). However, treatment with rapamycin inhibited this effect (DSV + Rapa: 28.35% ± 6.20 vs. DSV: 69.07 ± 0.16, *p* < 0.01, compared to DSV). Thus, our study suggests that rapamycin mediates its protective effect by inhibiting DSV-induced increased Snail expression and its nuclear translocation.

### 2.3. DSV Induces GSK-3 Phosphorylation and Rapamycin Prevents This Effect

**Figure 3:**
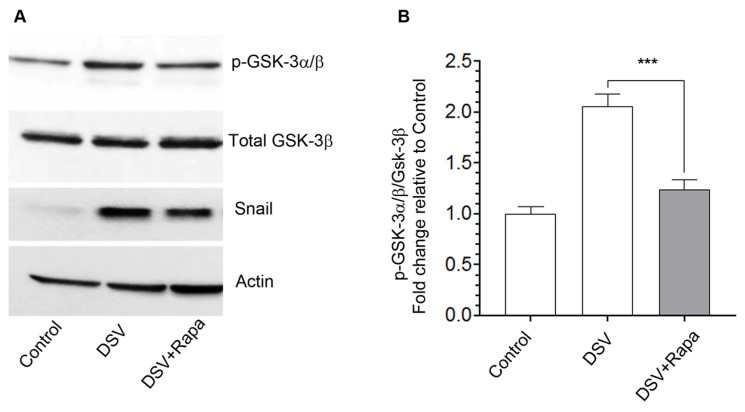
DSV induces GSK-3 phosphorylation and rapamycin prevents this effect. (**A**) Representative Western blots showing phospho-GSK-3α/β, total GSK-3β, and Snail expression in polarized cells infected with DSV with or without rapamycin pretreatment. Actin was used as a loading control. (**B**) Quantification of phospho-GSK-3α/β bands, normalized to total GSK-3β, and expressed relative to control using ImageJ/Fiji. Data represent mean ± SEM from at least three independent experiments. *** *p* < 0.001.

Stability of Snail and its nuclear localization is critical for its function. Snail is a target of a serine/threonine kinase, GSK-3, which mediates Snail phosphorylation at Ser-291, causing its nuclear exit and proteasomal degradation [20,21]. In contrast, inhibition of GSK-3α/β via phosphorylation at Ser-21/9 residues, respectively, inhibits its activity, thus allowing Snail activation. We tested whether DSV-induced increase in Snail occurred in conjunction with GSK-3 phosphorylation. Cells were infected with DSV and phosphorylation of GSK-3α/β was examined by Western blotting using an antibody that recognizes both the isoforms (Figure 3A). Values were normalized against total GSK-3β for quantification (Figure 3B). The phosphorylated GSK-3α band appeared more prominent when compared to the p-GSK-3β isoform in our experiment. We found that DSV caused a significant fold change increase in the levels of phospho-GSK-3 relative to the control, suggesting phospho-inactivation of GSK-3 (DSV: 2.06 ± 0.11 vs. control: 1.00 ± 0.06, *p* < 0.001, Figure 3A,B). As rapamycin is known to activate GSK-3, we tested whether rapamycin prevented DSV-mediated increase in p-GSK-3α/β. We found that in cells treated with DSV and rapamycin, the level of GSK-3 phosphorylation was significantly lower when compared to DSV-alone-infected cells (DSV + Rapa: 1.23 ± 0.09 vs. DSV: DSV: 2.06 ± 0.1, *p* < 0.001, compared to DSV, Figure 3B). Levels of Snail were similar to those observed in Figure 2A. These findings suggest that DSV increased Snail by concurrently inhibiting GSK-3 and rapamycin prevented these effects of DSV.

While rapamycin reverses DSV-mediated phosphorylation of GSK-3 and concomitantly inhibits Snail upregulation in response to DSV, further validation is needed to check whether Snail inhibition by rapamycin in response to DSV is dependent on GSK-3 and is not a mere parallel phenomenon. To provide supportive evidence that rapamycin may exert its protective effects on DSV-mediated outcomes in a GSK-3-dependent manner, we treated the cells with GSK-3 inhibitor SB216763 (SB) in the presence of rapamycin following infection with DSV (Figure S1). We tested whether SB reversed the inhibitory effect of rapamycin on DSV-induced increase in paracellular permeability by measuring FITC flux. We found that protective effects of rapamycin on DSV-induced flux were inhibited in the presence of SB (control: 144.6 ± 40.31; DSV 382.3 ± 9.64 vs. DSV + Rapa: 251.7 ± 36.77, *p* < 0.05 compared to DSV; DSV + Rapa + SB: 375.1 ± 80.48, *p* > 0.05 compared to DSV) (Figure S1A). We next tested whether SB inhibited the preventative effects of rapamycin on DSV-induced increased Snail protein expression (Figure S1B,C). Similarly to in Figure 2A, rapamycin prevented DSV-induced Snail protein expression (control: 1.0; DSV: 5.20 ± 1.02 vs. DSV + Rapa: 2.59 ± 0.56, *p* < 0.05 when compared to DSV). However, when SB was added along with rapamycin, it prevented the inhibitory effects of rapamycin, and Snail levels were observed to be comparable to those with DSV infection alone (DSV: 5.20 ± 1.02; DSV + Rapa + SB: 5.47 ± 0.58, *p* > 0.05 compared to DSV). Next, we analyzed the effects of SB on rapamycin-mediated protection of DSV-induced GSK-3 phosphorylation (Figure S1B,D). Cells were treated with rapamycin in the presence or absence of SB followed by infection with DSV. Fold change levels in the ratio of p-GSK-3α/β/total-GSK-3β were analyzed by Western blotting, and values were normalized against the control. DSV induced phosphorylation of GSK-3, and rapamycin prevented this effect (control: 1.0; DSV: 2.39 ± 0.28 vs. DSV + Rapa: 1.41 ± 0.17, *p* < 0.05 when compared to DSV). However, in cells treated with SB and rapamycin, p-GSK-3 levels were seen to be comparable to DSV (DSV: 2.39 ± 0.28; DSV + Rapa + SB: 2.24 ± 0.32, *p* > 0.05 when compared to DSV). SB also reversed the inhibitory effects of rapamycin on DSV-induced nuclear translocation of Snail (Figure S1E) (DSV: 47.48 ± 3.85 vs. DSV + Rapa: 20.53 ± 4.94, *p* < 0.001 compared to DSV; DSV + Rapa + SB: 53.83 ± 1.93, *p* > 0.05, compared to DSV). These findings, while not definitive in establishing causality, provide supportive evidence that GSK-3 activity is required for rapamycin’s protective effects against DSV-induced Snail activation and increased barrier permeability.

### 2.4. Inhibition of Proteasomal Degradation Negates the Protective Effect of Rapamycin on DSV-Induced Increased Permeability

**Figure 4:**
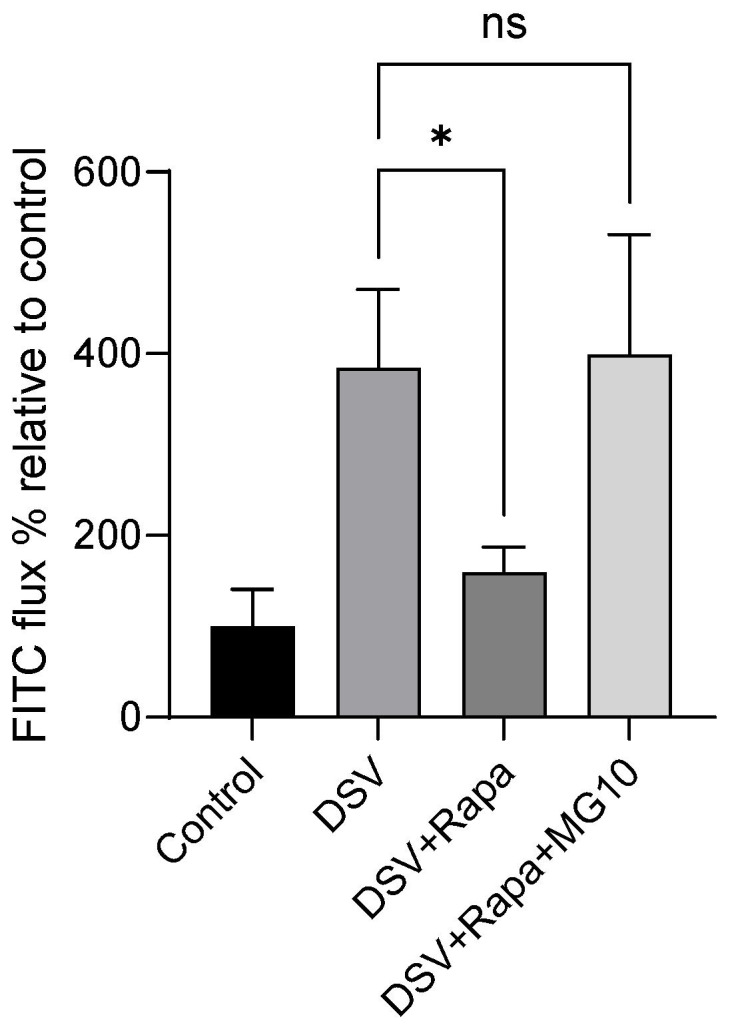
Inhibition of proteasomal degradation prevents the protective effect of rapamycin on DSV-induced increased permeability. Caco-2 cells were treated with rapamycin in the presence or absence of the proteasome inhibitor MG132 followed by DSV infection. Barrier permeability was assessed by measuring apical-to-basolateral FITC-dextran flux. Data represent percent FITC flux relative to uninfected control cells. Bars represent mean ± SEM from at least three independent experiments. Statistical analysis was performed using One-way Anova followed by multiple comparison with Dunnett’s test, comparing treatment groups with DSV group. * *p* < 0.05, ns: non-significant.

Lastly, we asked whether protective effects of rapamycin on DSV-induced FITC flux could be reversed by inhibiting proteasomal activity since GSK-3 mediates proteasomal degradation of Snail. For this, cells were pretreated with MG132, a well-known inhibitor of proteasomal degradation [22]. Rapamycin prevented DSV-induced increase in FITC flux when compared to DSV alone (DSV + Rapa: 159.5 ± 27.74 vs. DSV: 384.3 ± 86.30, *p* < 0.05, Figure 4). However, in the presence of MG132, rapamycin failed to inhibit DSV-induced FITC flux, and the flux in the presence of MG132 was comparable to DSV alone (DSV + Rapa + MG: 399.3 ± 131.7 vs. DSV: 384.3 ± 86.30, *p* > 0.05).

## 3. Discussion

DSV causes an increase in paracellular permeability in polarized intestinal Caco-2 cells by upregulating zinc finger nuclear transcription factor Snail, which is responsible for causing mislocalization of tight junction protein Occludin, leading to intestinal barrier dysfunction, as reported in our previous study [2]. In this study, we report that GSK-3, a negative regulator of Snail, was inhibited by DSV and that rapamycin, an mTOR inhibitor, prevented this phenomenon and ameliorated DSV-induced increased Snail expression as well as its nuclear translocation and DSV-induced increased barrier permeability. We also show supporting data demonstrating that rapamycin prevents DSV-induced effects by maintaining GSK-3 activity, at least in part, by using a specific GSK-3 inhibitor, SB21673. Moreover, blocking proteasomal degradation using MG-132 further prevents the protective effects of rapamycin on DSV-induced increased intestinal permeability.

GSK-3 is a highly conserved serine/threonine kinase with two isoforms, GSK-3α and GSK-3β, that control multiple signaling pathways and cellular functions such as cell proliferation, Wnt signaling, and glucose metabolism. Typically, GSK-3 exerts its effect by phosphorylating various substrates [23]. Dysregulation of GSK-3 is found in several diseases such as cancer, neurodegenerative diseases and inflammation [24]. GSK-3 regulates over 100 targets including the zinc finger transcription factor Snail [8]. GSK-3 inhibits Snail transcription in epithelial cells by inhibiting NFκB activity [25]. GSK-3 is also known to inhibit and de-stabilize Snail by phosphorylating it, causing β-Trcp-mediated Snail ubiquitination and degradation mainly by the proteasomal pathway [9]. Conversely, inhibiting GSK-3 activity via its phosphorylation causes increased stability of Snail and its nuclear import [12,13]. In this study, we found that DSV caused an increase in GSK-3 phosphorylation, thus inhibiting its activity and promoting Snail nuclear translocation.

The regulatory role of GSK-3 on Snail has mainly been studied in the context of epithelial-to-mesenchymal transition (EMT) and metastasis, where GSK-3 appears to play a protective role [9,26]. While Snail is extensively studied in EMT and cancer, we and others have published findings indicating that Snail is involved in causing increased intestinal permeability [2,27,28,29] and in disrupting the blood–brain barrier in response to a pathogen [30]. Our findings in this study suggest that GSK-3 may play a protective role in maintaining intestinal barrier function by inhibiting Snail in response to DSV. GSK-3 is an important player in many diverse cellular pathways, where its activity could be beneficial or harmful, depending on the context. Our data demonstrating a protective role of GSK-3 in barrier function is in agreement with some studies and in contradiction with others. A protective role of GSK-3 was demonstrated by Severson et al., showing that GSK-3 regulated apical junction proteins in polarized intestinal and kidney epithelial cells and that inhibition of GSK-3 resulted in increased barrier permeability with decreased expression of AJ proteins Occludin, Claudin-1 and E-cadherin [7]. In another study, human beta defensin (hBD) treatment increased the tight junction-related barrier and decreased paracellular flux in keratinocytes in a GSK-3-dependent manner [31]. Demonstrating a detrimental role of GSK-3 is the work by Ramirez et al., showing that inhibition of GSK-3 improved barrier integrity in primary brain microvascular endothelial cells (BMVECs) and treatment with GSK-3 inhibitors increased Occludin and Claudin-5 protein stability [32]. Similarly, it was found that degradation of GSK-3 by glucocorticoids increased transepithelial electrical resistance (TEER) in Con8 rat mammary epithelial tumor cells [33]. It is possible that these differential roles of GSK-3 may be dependent on the cell type, signaling pathways, the type of stimulus and the overall experimental setting and disease context. Nonetheless, dissecting the role of GSK-3α/β in intestinal barrier permeability in response to a pathobiont such as *Desulfovibrio*, an SRB that plays a contributory role in several diseases, is important in understanding and identifying unique cellular pathways that may be targeted to mitigate the harmful effects of *Desulfovibrio*.

How DSV causes phospho-inactivation of GSK-3 remains unknown. We have recently shown that DSV activates the pro-inflammatory PI3k/Akt pathway in macrophages. DSV activated PI3k/Akt in a toll-like receptor-2 (TLR-2) manner, which further induced pro-inflammatory TNF-α and inducible nitric oxide synthase (iNOS) production in response to DSV [34]. The PI3k/Akt pathway, a major signal transduction pathway, regulates many cellular functions involved in differentiation, metabolism, immunity and survival by orchestrating a vast number of downstream signaling effector molecules including GSK-3. Akt, also known as protein kinase B (PKB), is activated downstream of PI3K and phosphorylates and inhibits GSK-3 function [35,36,37,38]. The outcome of this phospho-inactivation is dictated by the upstream signals and downstream factors that are regulated by GSK-3, depending on the cell type and the type of stimulus. Conversely, GSK-3 can also phosphorylate and inactivate Akt [39]. We speculate that DSV may inhibit GSK-3 (via its phosphorylation) in intestinal epithelial cells by activating the upstream PI3K/Akt pathway, further suggesting that the regulatory effect of PI3k/Akt on GSK-3 in response to DSV may have implications beyond inflammatory response.

Another possible mechanism by which DSV may inhibit GSK-3 activity is by inhibiting Protein Phosphatase 2A (PP2A), a serine/threonine phosphatase responsible for dephosphorylating GSK-3 and increasing its activity [40]. PP2A is negatively related to Snail in the context of EMT [41,42] and in regulation of epithelial tight junctions in Sertoli cells [43]. PP2A also dephosphorylates and inactivates Akt [44]. Thus, by potentially inhibiting PP2A, DSV may cause increased PI3k/Akt activity and downstream Akt-mediated phosphorylation and inactivation of GSK-3. A correlation of PP2A activation and decreased intestinal permeability is also observed in some studies of PI3k/Akt [45,46]. Thus, the PP2A axis may offer a putative mechanism of how DSV may induce GSK-3 inhibition, Snail activation and increased intestinal permeability.

DSV products such as H_2_S can also cause inhibition of GSK-3. The role of H_2_S in modulating GSK-3 is documented in several experimental conditions, with some studies indicating an inhibitory effect while others demonstrate an activating effect of H_2_S on GSK-3, depending upon the H_2_S donor and the disease context [47,48,49]. Similarly, the role of LPS in modulating GSK-3 activity has also been documented [50,51,52]. Thus, it will be important to investigate whether H_2_S and LPS produced by DSV are directly involved in the DSV-induced inhibition of GSK-3.

Rapamycin is a well-known mTOR inhibitor that has been documented for its protective effects on the intestinal tight junction barrier [14,15] and has also been shown to activate GSK-3β and suppress cancer cells [18,19]. Rapamycin was found to inhibit Snail expression in a GSK-3-dependent manner in various cancer models in vivo and in vitro [53]. Whether or not rapamycin mediates its modulatory effects on Snail and GSK-3 in intestinal barrier permeability in response to a commensal gut pathobiont such as sulfate-reducing bacteria has not been explored. Our study shows that treatment of intestinal epithelial cells with rapamycin prevented DSV-induced increase in paracellular permeability and DSV-induced Snail expression and its nuclear transport. Rapamycin also inhibited DSV-induced increased phosphorylation of GSK-3, thus increasing its activity. As GSK-3 facilitates proteasomal degradation of Snail [9], we found that by inhibiting proteasomal activity using MG132, rapamycin could no longer inhibit DSV-induced permeability, suggesting that rapamycin may mediate its protective effect via GSK-3. A positive regulatory effect of rapamycin on GSK-3 is not a universal phenomenon, and a few studies have reported no effects of rapamycin on GSK-3 activity in various experimental settings involving different cell types such as hepatocytes and myoblasts [54,55]. In another instance, it was found that rapamycin may block GSK-3 activation by one stimulus and not the other [56], suggesting that stimulatory effects of rapamycin on GSK-3 may depend on cell types and the type of stimulus.

How rapamycin prevents DSV-induced GSK-3 phospho-inactivation remains unknown. It is possible that rapamycin may inhibit a kinase downstream of mTOR that could be responsible for GSK-3 phosphorylation. One such example is that of P70S6 kinase, which was shown to constitutively phosphorylate GSK-3 and inhibit its activity [57]. We have previously reported that DSV activates P70S6 kinase downstream of the PI3k/Akt pathway [34], providing yet another potential mechanism of how DSV may inactivate GSK-3 via P70S6 activation. Since rapamycin inhibits S6 kinase in the mTOR pathway, it provides a putative mechanism of how rapamycin may override inactivation of GSK-3 by DSV. We showed that SB21673 prevented rapamycin from suppressing Snail induction and barrier permeability in response to DSV. While pharmacologic inhibition cannot establish causality on its own, the observation that SB21673 inhibited rapamycin’s protective effects provides functional support for the idea that rapamycin acts, at least in part, by maintaining GSK-3 activity. Future studies using genetic gain- or loss-of-function approaches will be needed to confirm these pathways.

In our previous study, we showed that intestinal alkaline phosphatase (IAP) inhibited DSV-induced increase in barrier permeability and Snail activation. It is currently unknown whether IAP can also overcome DSV-induced inhibition of GSK-3. A potential mechanistic link between IAP and rapamycin is that both induce autophagy [58,59]. Autophagy is known to promote degradation of Snail [60,61]. Whether autophagy acts as a downstream effector of IAP- or rapamycin-mediated protection via GSK-3 and Snail remains to be determined. As the Akt pathway inhibits GSK-3 and DSV induces Akt, it also remains to be explored whether rapamycin and IAP inhibit DSV-induced Akt activation. Further studies dissecting these interconnected pathways will be necessary to define how IAP and rapamycin may converge on the GSK-3/Snail axis to preserve epithelial barrier integrity. A limitation of this study is that our data demonstrates a correlation between Snail and GSK-3 pathways but lacks evidence to prove causality. Along these lines, a mechanism of how DSV may inhibit GSK-3 via phosphorylation and knowledge of whether GSK-3 inhibition is the cause of Snail upregulation and permeability are lacking. Similarly, the underlying mechanism of how rapamycin prevents these effects of DSV remains unknown.

Future investigations of the mechanisms underlying GSK-3 regulation by rapamycin as well as those establishing a causal relationship between GSK-3 and Snail in response to DSV are needed to further understand and identify novel pathways and therapeutic targets that may play a role in DSV-mediated intestinal permeability. Further, overexpression of GSK-3 could be utilized to directly examine whether restoration of GSK-3 activity could overcome DSV-induced Snail activation and barrier permeability. Moreover, studies examining additional epithelial cell lines, organoid models, and in vivo models will be important for determining whether the DSV–GSK-3–Snail pathway described here is cell type-specific or more widely conserved. In addition, investigating localization of TJPs such as Occludin would provide further insight into the protective role of rapamycin in preventing DSV-induced TJP dysfunction. Finally, testing the role of other mTOR inhibitors such as INK128 would also be important to determine whether the protective effect on epithelial barrier function is a broader consequence of mTOR pathway inhibition.

### Conclusions

In summary, this study demonstrated that DSV caused phospho-inactivation of GSK-3 in intestinal epithelial cells, that this correlated with an increase in the Snail transcription factor and increased intestinal permeability in response to DSV, and that all these effects could be overcome by rapamycin. These findings identify the GSK-3/Snail axis as a potential mechanism underlying SRB-induced barrier dysfunction and suggest that modulation of this pathway may represent a therapeutic strategy for preserving epithelial barrier integrity.

## 4. Materials and Methods

### 4.1. Cell Culture and Treatments

Human colonic epithelial cells, Caco-2 (ATCC, Manassas, VA, USA), were grown in DMEM containing 20% FBS (Thermo Fisher Scientific, Waltham, MA, USA) at 37 °C in a humidified incubator with 5% CO_2._ Cells were allowed to differentiate and polarize by seeding at a density of 5 × 10^5^ cells/well in 12-well 0.4 μm trans-well inserts for 3 weeks. Medium was replaced twice a week. Cells were infected with DSV at the apical surface at multiplicity of infection (MOI) 20 for 24 h. For rapamycin treatment, cells were incubated with either 50 nM rapamycin (Cell Signaling Technology (Danvers, MA, USA)) or DMSO vehicle at the apical and basolateral surface for 2 h followed by infection with DSV.

#### 4.1.1. *Desulfovibrio vulgaris* (DSV) Culture and Infection

*Desulfovibrio vulgaris* Hildenborough (ATCC 29579, Manassas, VA, USA) was grown anaerobically in Hungate tubes using Postgate’s organic liquid medium. Medium composition was as follows: 10.56 mM Na_2_SO_4_, 13.29 mM MgSO_4_, 4.12 mM L-Cysteine, 0.4% sodium lactate (60% syrup), 0.4% yeast extract, and 0.5% tryptone. Cultures were grown for 24 h in 5 mL aliquots at 37 °C. Bacteria were counted using a Quantom Tx cell counter (Logos Biosystems, Anyang-si, Republic of Korea) according to the company’s instructions. Polarized Caco-2 monolayers contained 1 × 10^6^ cells per well, so MOI 20 corresponded to 20 × 10^6^ bacteria per well. After counting, 1 mL of bacterial culture was collected and centrifuged, and the pellet was resuspended in 1 × PBS. The appropriate volume corresponding to 20 × 10^6^ bacteria was then added to the apical surface of the cells.

#### 4.1.2. FITC Flux

Paracellular permeability across the 3-week-old polarized Caco-2 cells grown in 12-well trans-well plates was assessed by measuring the movement of FITC-dextran across the intestinal layer as previously published [2].

#### 4.1.3. Western Blot

Cells were lysed in lysis buffer in the presence of protease and phosphatase inhibitors as previously described [2]. Briefly, cell lysis was carried out for 30 min at 4 °C with shaking. Lysates were centrifuged at 12,000 rpm for 5 min at 4 °C, supernatants collected, and protein concentration determined. Then, 50 µg of protein samples was run on SDS-PAGE (4–20% tris-glycine) and transferred to nitrocellulose membranes. Membranes were blocked in 5% milk in PBS-T (0.1%Tween 20) for 30 min followed by overnight incubation in antibodies against actin (Cell Signaling Technology: 4970) and Snail (Cell Signaling Technology: #3879), phosphorylated GSK-3a/b Ser 21/9 (9331) and total GSK-3b (12456). Antibodies were diluted as recommended by the manufacturer. Blots were incubated with secondary antibodies (Cell Signaling Technology: #7074) at room temperature for 1 h (dilution of 1:2000) and developed using enhanced Chemiluminescence HRP signal (Thermo Fisher Scientific: #34577).

#### 4.1.4. Immunofluorescence

Cells grown on trans-wells were processed for immunofluorescence as previously reported [2]. Briefly, cells were fixed with 4% paraformaldehyde and blocked in a blocking solution consisting of 5% FBS and 0.3% Triton-X 100. This was followed by incubation of both the apical and basolateral surface of the trans-wells with primary antibody against Snail1 (Cell Signaling Technology: 91131) at 4 °C. Cells were then washed with PBS and incubated with Alexa Flour-labeled secondary antibody for 2 h at room temperature. Imaging was done with an Olympus Fluoview FV1200 confocal microscope (Olympus Corporation, Tokyo, Japan).

#### 4.1.5. Statistical Analysis

All graphs were generated using GraphPad Prism 9 (GraphPad Software, San Diego, CA, USA). Data represents mean ± SEM from at least three independent experiments. Values were normalized to the control and presented as the percent change relative to the control set at 100%. Student’s *t*-test was utilized for statistical analysis when comparing two groups. For comparing more than two groups, One-way Anova followed by Dunnett’s test for multiple comparisons was utilized. DSV was used as a reference group for all analyses. *p* values < 0.05 were considered statistically significant.

## Data Availability

The original contributions presented in this study are included in the article/Supplementary Material. Further inquiries can be directed to the corresponding author.

## Supplementary Materials

The following supporting information can be downloaded at https://www.mdpi.com/article/10.3390/microorganisms14040781/s1, Figure S1. Inhibition of GSK-3 attenuates protective effects of Rapamycin. Polarized Caco-2 cells were treated with either rapamycin alone (50 nM for 2 h) or with rapamycin in combination with SB216763 (5 mM), (a GSK-3 inhibitor) followed by DSV infection for 24 h. (**A**) Cells were incubated for 1 h with 40 μL 4 kDa FITC-Dextran (25 mg/mL) added to the apical surface. After 1 h, 100 μL of the medium from basolateral side was removed and analyzed for FITC fluorescence, using excitation and emission at 485 nm and 520 nm, respectively. Graph represents Mean ± SEM of percent values of FITC-flux normalized to control, from at least 3 independent experiments. (**B**) Cells were treated with rapamycin (50nM ) alone or in combination with SB for 2 hrs followed by DSV infection for 24 hours. Cells were then processed for western blotting to detect the protein levels of Snail, phospho-GSK-3, and total GSK-3. Actin was used a loading control. (**C**) Blot were quantified using Fiji Image J. Ratio of Snail/Actin was analyzed and values were normalized to control cells. * *p* < 0.05. (**D**) Blot were quantified using Fiji Image J. Ratio of p-GSK-3/total GSK-3 was analyzed and values were normalized to control cells. Values were normalized to uninfected control. Graph represents Mean ± SEM from at least 3 independent experiments. * *p* < 0.05. (**E**) Quantification of immunofluorescence. Cells were treated with rapamycin alone or together with SB before DSV challenge. Cells were imaged for detection of nuclear Snail similar to Figure 2C. Graph represents Mean ± SEM of percentage of cells positive for nuclear Snail staining. *** *p* < 0.001. * *p* < 0.05.

## Abbreviations

The following abbreviations are used in this manuscript:

DSV	*Desulfovibrio vulgaris*
GSK-3	Glycogen synthase kinase -3
FITC	Fluorescein isothiocyanate
mTOR	Mammalian target of rapamycin
TEER	Transepithelial electrical resistance
