# Supplementary material for: Rapamycin Prevents Sulfate-Reducing Bacteria-Induced Effects on Snail and GSK-3 and Impaired Intestinal Barrier
Source: Microorganisms. 2026 Mar 30;14(4):781. doi: 10.3390/microorganisms14040781 (PMC13118803; doi:10.3390/microorganisms14040781)
Supplement: Supplementary file 1 [file microorganisms-14-00781-s001.zip › microorganisms-4194331-supplementary.pdf]

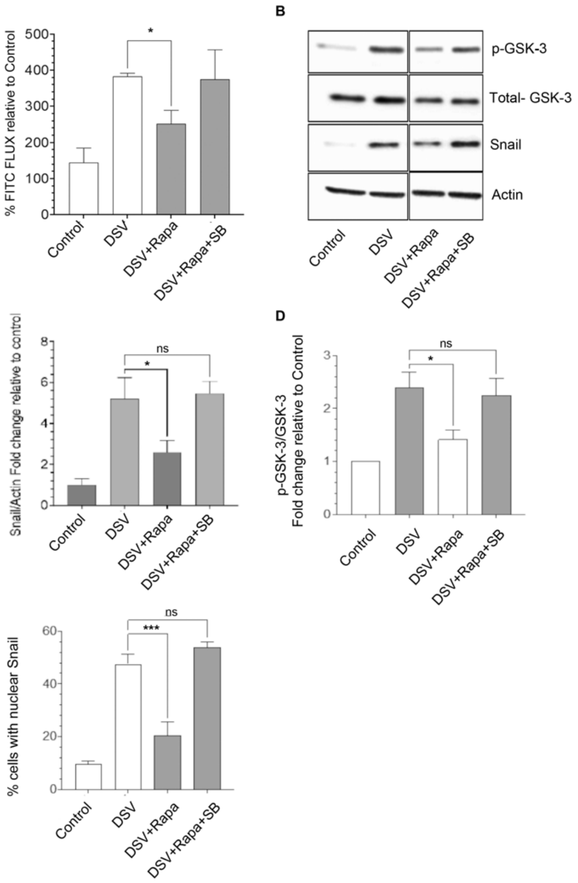

Figure S1. Inhibition of GSK-3 attenuates protective effects of Rapamycin. Polarized Caco-2 cells were treated with either rapamycin alone (50 nM for 2 h) or with rapamycin in combination with SB216763 (5 mM), (a GSK-3 inhibitor) followed by DSV infection for 24 h. (A) Cells were incubated for 1 h with 40 μL 4 kDa FITC-Dextran (25 mg/mL) added to the apical surface. After 1 h, 100 μL of the medium from basolateral side was removed and analyzed for FITC fluorescence, using excitation and emission at 485 nm and 520 nm, respectively. Graph represents Mean±SEM of percent values of FITC-flux normalized to control, from at least 3 independent experiments. (B) Cells were treated with rapamycin (50nM) alone or in combination with SB for 2 hrs followed by DSV infection for 24 hours. Cells were then processed for western blotting to detect the protein levels of Snail, phospho-GSK-3, and total GSK-3. Actin was used a loading control. (C) Blot were quantified using Fiji Image J. Ratio of Snail/Actin was analyzed and values were normalized to control cells. * $p < 0.05$. (D) Blot were quantified using Fiji Image J. Ratio of p-GSK-3/total GSK-3 was analyzed and values were normalized to control cells. Values were normalized to uninfected control. Graph represents Mean±SEM from at least 3 independent experiments. * $p < 0.05$. (E) Quantification of immunofluorescence. Cells were treated with rapamycin alone or together with SB before DSV challenge. Cells were imaged for detection of nuclear Snail similar to Figure 2C. Graph represents Mean ± SEM of percentage of cells positive for nuclear Snail staining. *** $p < 0.001$. * $p < 0.05$.
